# Dynamic Spatial-Temporal Memory Augmentation Network for Traffic Prediction

**DOI:** 10.3390/s24206659

**Published:** 2024-10-16

**Authors:** Huibing Zhang, Qianxin Xie, Zhaoyu Shou, Yunhao Gao

**Affiliations:** 1 Guangxi Key Laboratory of Trusted Software, Guilin University of Electronic Technology, Guilin 541004, China; zhanghuibing@guet.edu.cn (H.Z.);; 2 School of Information and Communication, Guilin University of Electronic Technology, Guilin 541004, China; guilinshou@guet.edu.cn; 3 Institute of Geotechnical and Underground Engineering, Beijing University of Technology, Beijing 100124, China

**Keywords:** smart city, traffic flow prediction, multiple self-attention mechanism, graph convolutional network, meta-knowledge learning

## Abstract

Traffic flow prediction plays a crucial role in the development of smart cities. However, existing studies face challenges in effectively capturing spatio-temporal contexts, handling hierarchical temporal features, and understanding spatial heterogeneity. To better manage the spatio-temporal correlations inherent in traffic flow, we present a novel model called **D**ynamic **S**patio-**T**emporal **M**emory-**A**ugmented **N**etwork (**DSTMAN**). Firstly, we design three spatial–temporal embeddings to capture dynamic spatial–temporal contexts and encode the unique characteristics of time units and spatial states. Secondly, these three spatial–temporal components are integrated to form a multi-scale spatial–temporal block, which effectively extracts hierarchical spatial–temporal dependencies. Finally, we introduce a meta-memory node bank to construct an adaptive neighborhood graph, implicitly representing spatial relationships and enhancing the learning of spatial heterogeneity through a secondary memory mechanism. Evaluation on four public datasets, including METR-LA and PEMS-BAY, demonstrates that the proposed model outperforms benchmark models such as MTGNN, DCRNN, and AGCRN. On the METR-LA dataset, our model reduces the MAE by 4% compared to MTGNN, 6.9% compared to DCRNN, and 5.8% compared to AGCRN, confirming its efficacy in traffic flow prediction.

## 1. Introduction

The transportation system, as a key component of modern cities, directly influences the efficiency of urban operations through effective traffic management, route planning, and congestion mitigation [1,2,3]. With the accelerated advancement of smart city construction, higher demands are being placed on the development of Intelligent Transportation Systems (ITSs). Traffic flow prediction serves as a fundamental basis and driving force in building urban ITSs. Therefore, thoroughly modeling the spatio-temporal relationships within the transportation system and comprehensively capturing the complex, dynamic spatio-temporal dependencies for traffic flow prediction have become a core issue in ITS research. Given the spatio-temporal complexity of transportation systems, addressing traffic flow prediction remains a critical challenge, particularly in capturing **spatial heterogeneity**, **spatial**–**temporal context**, and **multi-scale temporal dependencies** [4].

The challenges in spatial–temporal modeling for traffic flow prediction are manifold: (1) the spatial heterogeneity of traffic patterns due to regional functional differences. For example, spatial heterogeneity in traffic patterns can be caused by regional functional differences. Commercial areas may experience traffic peaks at specific times during weekdays, while residential areas may encounter traffic surges in the morning and evening. These differences result in distinct traffic flow distributions. Capturing this spatial heterogeneity through external data is challenging due to the functional variations between areas; (2) the complex spatial–temporal dependencies arising from residents’ spatial locations and travel patterns, rather than relying solely on temporal dependencies. Factors such as residents’ daily travel habits, urban planning, and transportation infrastructure all play a significant role in the spatio-temporal distribution of traffic flow; (3) the multi-scale temporal correlations under varying traffic conditions (e.g., congested, free-flowing, etc.). For example, over short periods of time, traffic flows may change rapidly due to unexpected events such as traffic accidents or weather changes. Conversely, over longer time scales, cyclical patterns such as differences between weekday and weekend flows can be observed.

Currently, the synergistic integration of graph and sequence models is primarily employed for spatial–temporal modeling. For instance, ref. [5] employs adaptive graph learning to mitigate the bias introduced by a priori knowledge, but the consideration of spatial heterogeneity remains insufficient. In another study, ref. [6] utilized parametric embedding combined with simple MLPs to achieve efficient prediction performance; however, this approach weakened the effect of multi-scale spatial–temporal dependence. Additionally, ref. [7] incorporated complex spatial–temporal modeling components, which, despite improving performance, are computationally expensive.

To address the aforementioned problems, this paper proposes a prediction model for a **D**ynamic **S**patial–**T**emporal **M**emory **A**ugmentation **N**etwork **(DSTMAN)** to effectively capture spatial heterogeneity, spatial–temporal context, and multi-scale temporal dependency. A diverse set of spatial–temporal embeddings are designed to comprehensively encode background information such as time and overall spatial state. Additionally, a meta-node repository is defined to encode typical feature archetypes in traffic scenarios during the graph structure learning phase, allowing for the adaptive learning of node neighborhoods. Furthermore, a secondary memory enhancement module is introduced prior to the model output layer to learn spatial heterogeneity by utilizing the similarity of the meta-node repository through a retrieval attention mechanism. The main contributions of this paper are as follows:

- We propose a dynamic spatial–temporal memory augmentation network for traffic prediction tasks. This model comprehensively encodes spatial–temporal contextual information, captures both global and local correlations of traffic data using various convolutional modules, and adaptively learns spatial heterogeneity. As a result, it efficiently captures spatial–temporal contexts, multi-scale temporal dependencies, and spatial heterogeneity.
- We employ cascaded spatial–temporal blocks to construct the model backbone, comprising multi-head temporal attention modules, diffusion graph convolution modules, and multi-scale convolutional layers. These components are designed to learn and capture multi-scale spatial–temporal information.
- We innovatively design a meta-node bank to capture the representative feature prototypes of typical samples without auxiliary data. This meta-node bank is leveraged to construct a meta-graph learner used in the diffusion graph convolution layers. Additionally, we strengthen the modeling of spatial heterogeneity by applying a secondary retrieval-based attention mechanism that exploits the similarity to the meta-nodes.

## 2. Related Works

Traffic prediction is a key research area in spatio-temporal data mining. Early approaches, such as [8,9], modeled traffic data as independent time series. However, these traditional statistical techniques have limitations in capturing complex non-linear relationships. Recurrent neural networks (e.g., GRU [10], LSTM [11]) and Transformer [12] excel in capturing the temporal dynamics of traffic signals, while graph convolutional networks [13] have been successful in exploiting topological structures. For instance, ref. [14] combines gated linear cells and ChebNet operators for traffic prediction, and ref. [15] uses diffusion graph convolution to jointly model spatial and temporal dependencies. Despite these advancements, many methods rely on pre-defined graph structures with a priori assumptions that limit the modeling of spatial dependencies. To address this limitation, refs. [5,16,17] designed adaptive adjacency matrices to learn potential spatial connections, significantly improving performance. In recent years, studies such as [6,18,19,20,21] have demonstrated innovations in spatio-temporal modeling [22]. By integrating the conditional neural process and the memory network, the accuracy and reliability of traffic prediction are effectively improved. However, these studies primarily focus on spatio-temporal correlation, neglecting issues related to spatio-temporal context and spatial heterogeneity. Specifically, most existing studies emphasize capturing temporal and spatial dependencies but do not adequately consider spatio-temporal contextual information in transport systems. Furthermore, traffic patterns in different regions may vary significantly due to regional functional differences (e.g., commercial, residential, industrial areas), and this spatial heterogeneity has not been sufficiently modeled in existing approaches.

In recent years, meta-knowledge learning and inference have been able to capture the relational and structural patterns of related tasks even with limited training data, enabling models to adapt quickly to new tasks. For instance, ref. [17] introduced a node-adaptive parameter learning mechanism that dynamically generates node-specific model parameters by learning embedded representations of graph structures. Similarly, ref. [23] dynamically generated LSTM cell parameters based on the current input context through a shared Meta-LSTM. Additionally, ref. [24] constructed a meta-node bank to capture the heterogeneity between different spatial entities through adaptive parameterization. Furthermore, ref. [25] proposed a meta-parameter learning scheme for joint spatio-temporal dimensions running in a Vanilla Transformer. In another study, ref. [26] dynamically configured task-specific model parameters based on meta-knowledge, which improved the model generalization capabilities and adaptability in different cities. Moreover, ref. [27] utilized a city’s points of interest and road network information as meta-knowledge in a traffic prediction task to customize model parameters. These studies demonstrate the potential of meta-knowledge learning in traffic prediction, but there are some shortcomings. Firstly, most of the work focuses on the dynamic tuning of model parameters, while there is less research on the acquisition and representation of meta-knowledge. Secondly, existing methods are still inadequate for modeling heterogeneity in complex spatio-temporal environments.

## 3. Method

Traffic forecasting is the practice of predicting future traffic using a given sequence of historical observations along with an auxiliary prior graph. The traffic network can be simplified as a graph $G=\left(V,E,A\right)$. Here, *V* is the set of nodes, which represents *N* = ∣*V*∣ traffic sensors for a distribution of observations. *E* denotes the set of edges, and *A* denotes the adjacency matrix that characterizes the relationships between different nodes. Assuming that each time series has a total of *T* timestamps, we can represent the signals of all nodes on *G* as a three-dimensional feature matrix $X\in {\mathbb{R}}^{T\times N\times D}$, where *D* is the feature dimension. Similarly, $X_{t}^{\left(i\right)}$ denotes the traffic features collected from the *i*-th sensor at the *t*-th timestamp.

The objective of the forecasting task is to establish a mapping $F_{\theta }\left(\cdot \right)$ from historical observations to future data. It is formally defined as follows:

(1) $$\left[X^{\left(t-P+1\right)},\cdots ,X^{\left(t-1\right)},X^{\left(t\right)};G\right]\overset{F_{\theta }\left(\cdot \right)}{\to }\left[{\hat{X}}^{\left(t+1\right)},{\hat{X}}^{\left(t+2\right)},\cdots ,{\hat{X}}^{\left(t+Q\right)}\right]$$

where *θ* denotes the learnable model parameters, *P* and *Q* denote the lengths of the historical and predicted sequences, respectively.

**Figure 1:**
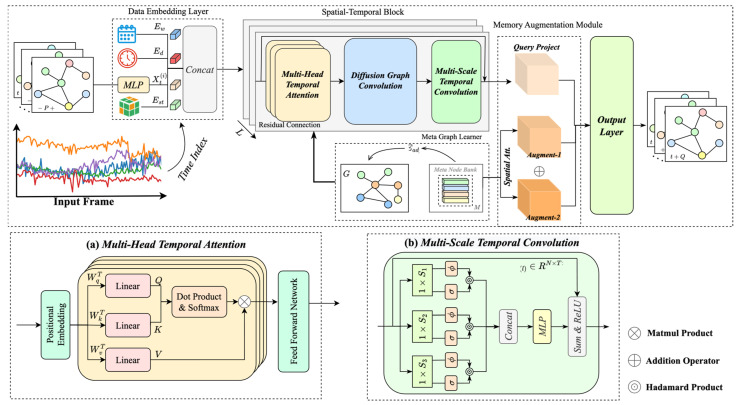
Overall architecture of DSTMAN.

The Dynamic Spatial–Temporal Memory Augmentation Network (DSTMAN) proposed in this paper is illustrated in Figure 1. The data embedding layer is designed with a variety of learnable embeddings that assign temporal and spatial units to markers with day and week cycles. Secondly, the spatio-temporal block consists of a multi-head temporal attention module, a diffusion graph convolution module, and a multi-scale convolution layer for learning and capturing multi-scale spatio-temporal information. Finally, the meta-node bank captures representative feature prototypes of typical samples to enhance the modeling of spatial heterogeneity.

### 3.1. Data Embedding Layer

To enable the model to recognize and distinguish spatio-temporal contexts, it is necessary to encode specific temporal and spatial states when mapping low-dimensional features to high-dimensional representations. We design various learnable embeddings in the data embedding layer that assign tokens to temporal and spatial units with periods of one day and one week.

Firstly, we project the original features through a fully connected layer to obtain the high-dimensional feature embedding $E_{f}=FC\left(X_{t-T+1:t}\right),\;E_{f}\in {\mathbb{R}}^{T\times N\times d_{f}}$. We then construct two learnable temporal embedding dictionaries, denoted as $T_{d}\in {\mathbb{R}}^{N_{w}\times d_{t}}$ and $T_{t}\in {\mathbb{R}}^{N_{t}\times d_{t}}$, which encode the day-of-week and time-of-day temporal contexts, respectively. In this case, $N_{w}$ represents the number of days in a week, while $N_{t}$ denotes the number of time slices within a day. In practice, we extract temporal index representations $D_{t}\in {\mathbb{R}}^{T}$ and $W_{t}\in {\mathbb{R}}^{T}$ from the time series frame $X_{t-T+1:t}$ and use these slices to retrieve the corresponding day-of-week embeddings $E_{W}\in {\mathbb{R}}^{T\times d_{t}}$ from $T_{d}$ and time-of-day embeddings $E_{d}\in {\mathbb{R}}^{T\times d_{t}}$ from $T_{t}$, respectively. By concatenating and broadcasting the retrieved two embeddings, we obtain the periodicity embedding $E_{p}\in {\mathbb{R}}^{T\times N\times 2d_{t}}$, which encodes the temporal periodicity information for the traffic time series. The motivation of the temporal embeddings is to encode representations that can implicitly identify and explicate the temporal heterogeneity exhibited across multiple temporal scales.

Secondly, we also design a spatial–temporal mixed embedding $E_{st}$ to capture the cascading influences across different temporal sequences (i.e., traffic sensors) along the chronological order. $E_{st}\in {\mathbb{R}}^{T\times N\times d_{s}}$ is initialized directly as an embedding vector, rather than being extracted from a learned dictionary, as is the case for the temporal embeddings. The aim of introducing the $E_{st}$ is twofold: on the one hand, it accounts for the pattern differences along the temporal order; on the other hand, it reflects the potential service-oriented or functional influences across different nodes, thereby encoding the context information associated with the spatial locations.

Lastly, we sum up $E_{pe}$ and concatenate the above embeddings to obtain spatial–temporal representation *H* as follows:

(2) $$H=\left(E_{f}+E_{pe}\right)\left|\left|E_{p}\right|\right|E_{st}$$

where $E_{pe}$ is the cosine position encoding in the next attention module, and $H\in {\mathbb{R}}^{T\times N\times d_{h}},d_{h}=d_{f}+2d_{t}+d_{s}$.

### 3.2. Multi-Head Temporal Attention Module

In the spatial–temporal module structure shown in Figure 1a, a multi-head temporal attention mechanism and a multi-scale temporal convolutional layer are combined. The former contains a location coding layer, a multi-head attention mechanism, and a feed-forward network to enhance the model’s ability to capture long-range temporal dependencies in the input sequence. The input representation $H\in {\mathbb{R}}^{T\times N\times d_{h}}$ is fed into this module, already equipped with position coding in the data embedding layer to demonstrate the relative position of the sequence. This is particularly important for traffic prediction, as traffic flows are significantly time-dependent and periodic. The query, key, and value matrices are then generated using $H^{\left(i\right)}$ of the i-th block. The formula is as follows:

(3) $$Q=H^{\left(i\right)}W_{q}^{T},K=H^{\left(i\right)}W_{k}^{T},V=H^{\left(i\right)}W_{\upsilon }^{T}$$

(4) $$Attention\left(Q,K,V\right)=softmax\left(\frac{QK^{T}}{\sqrt{d_{k}}}\right)V\;$$

where $W_{q}^{T},W_{k}^{T,}$ and $W_{v}^{T}$ are learnable projection parameters. The query matrix *Q* is multiplied by the transpose of the key matrix *K*, then scaled and normalized to obtain the attention distribution at each time step. Here, $QK^{T}$ denotes the dot product of the query matrix and the transpose of the key matrix, and $d_{k}$ is the dimension of the key matrix. Finally, the attention score matrix is weighted on the value matrix *V* to generate an implicit feature representation. This mechanism is effective in capturing the long-range dependencies and temporal dynamic features of the input sequence.

To enable the model to jointly attend to information from different representation subspaces as well as diverse temporal patterns, outputs from multiple heads are concatenated and linearly transformed using the projection matrix $W^{O}$ to generate the final result. The formulae are as follows:

(5) $$\begin{array}{c} MultiHead\left(Q,K,V\right)=Concat\left(head_{1},head_{2},\cdots ,head_{h}\right)W^{O}\\ head_{i}=Attention\left(\hat{H}W_{i}^{Q},\hat{H}W_{i}^{K},\hat{H}W_{i}^{V}\right),i\in \left[1,h\right] \end{array}\;$$

where $W_{i}^{Q},W_{i}^{K},and\;W_{i}^{V}\in {\mathbb{R}}^{d_{m}\times d_{k}}$ are the linear projection parameters of each attention head, and $d_{k}=h\times d_{m}$, where *h* is the number of attention heads. The multi-head attention mechanism captures features at different time steps and in different traffic patterns through parallel attention heads, allowing the model to understand the complexity of traffic flow data more comprehensively. Eventually, the feed-forward neural network receives the output from the multi-head attention module and processes it further.

### 3.3. Diffusion Graph Convolutional Module

To further enhance the performance of node embeddings generated from spatio-temporal graphs in modeling spatial heterogeneity, this paper introduces a meta-graph **learner** that enables the model to extract and store representative spatial features from observation samples, reflecting important patterns and relationships in the transport network. The core idea is to construct a meta-node bank that stores representative spatial features extracted from the observation samples. These meta-nodes serve as a pattern reference to facilitate the matching and recognition of potential spatial heterogeneity in the graph structure learning process.

The meta-node bank can be represented as $\Phi \in {\mathbb{R}}^{M\times d}$, where *M* denotes the number of meta-memory nodes and *d* denotes the feature dimension of each meta-node. The relationship between the spatial features of different traffic nodes and the features in the meta-node bank is learned by performing a matrix computation between the meta-node bank *Φ* and the adaptive weighting factor $W_{E}$ to generate the memory-augmented node embedding $W_{adp}$, which is then used to construct the meta-graph learner. The equations are represented as follows:

(6) $$\left\{\begin{array}{c} E_{adp}=W_{E}\cdot \Phi \\ \hat{A}=softmax\left(ReLU\left(E_{adp}\cdot E_{adp}^{T}\right)\right) \end{array}\right.\;$$

where $W_{E}\in {\mathbb{R}}^{N\times M}$ denotes the adaptive weighting factor and $E_{adp}\in {\mathbb{R}}^{N\times d}$ denotes the meta-memory-augmented embedding. Here, a meta-graph $\hat{A}\in {\mathbb{R}}^{N\times N}$ is generated, which can effectively represent the complex spatial relationships in the traffic network. Feeding it back into the diffusion graph convolution module provides more accurate structural information for subsequent graph convolution operations.

Given the directionality of traffic signal diffusion, we perform bidirectional random walks on the meta-graph to capture multi-hop spatial correlations. The formula for the diffusion graph convolutional module is as follows:

(7) $$g_{\theta }\times_{G}\left(H,\hat{A}\right)=\overset{K}{\underset{k=0}{\sum }}\;\;{\hat{A}}_{f}^{k}HW_{k1}+{\hat{A}}_{b}^{k}HW_{k2}\;$$

where *k* denotes the order of the diffusion convolution. ${\hat{A}}_{f}=\frac{A}{rowsum\left(A\right)}\;\mathrm{and}\;{\hat{A}}_{b}=\frac{A^{T}}{rowsum\left(A^{T}\right)}$ are the respective forward and backward transition matrices. $W_{k1}$ and $W_{k2}$ are the learnable weight parameters.

### 3.4. Multi-Scale Temporal Convolution Module

Traffic flow data are multi-scale time-dependent, including both sudden changes over short periods of time and periodic patterns over long periods of time. Therefore, a multi-scale time convolution module (shown in Figure 1b) is designed to capture sequential patterns in time series data using 1D convolution filters with different receptive fields. By introducing 1D convolution filters with different receptive fields, the model is able to capture various time series features. Each standard convolution in the module is followed by a hyperbolic tangent function, which acts as a filter, and a Sigmoid function, which acts as a gating mechanism to control the flow of information conveyed by the convolution. The fusion of multi-scale temporal features helps to improve the model’s ability to perceive the complex time dependence of the road network.

Let the hidden layer representation fed into the temporal module be denoted as $H^{\left(i\right)}\in {\mathbb{R}}^{N\times T\times C}$. We apply standard one-dimensional convolutional kernels of size $1\times S_{i}$ to filter $H^{\left(i\right)}$ along the time axis. The 1D convolution kernel is defined as $\Gamma \left(x\right)\in {\mathbb{R}}^{S_{i}\times C\times 2C},$ and the filtered signal representation is ${\hat{H}}^{\left(i\right)}=\Gamma \left(x\right)\times H^{\left(i\right)}$, where ${\hat{H}}_{\left(l\right)}\in {\mathbb{R}}^{N\times \left(T-S_{i}+1\right)\times 2C}$. The equation of the gated temporal unit is depicted as follows:

(8) $$GConv_{1\times S_{i}}=\phi \left(P\right)⊙\sigma \left(Q\right)\;$$

where *P* and *Q* are the first half and the second half of ${\hat{H}}^{\left(i\right)}$ relative to the channel dimension. $\phi \left(\cdot \right)$ and $\sigma \left(\cdot \right)$ represent the tanh and Sigmoid functions, respectively.

In the practical implementation, we construct a multi-resolution temporal convolutional module by employing a set of diverse convolutional kernel sizes as follows: 1 × 2, 1 × 3, 1 × 5, and 1 × 7. This design choice is motivated by the ability of these kernel sizes to compose a variety of temporal coverage periods maximally, rendering the module well suited for capturing short-term signal patterns; that is defined as below:

(9) $$Z^{\left(i\right)}=Concat\left(GConv_{1\times 2},GConv_{1\times 3},GConv_{1\times 5},GConv_{1\times 7}\right)$$

where $Z^{\left(i\right)}$ is the output of the four filters concatenated across the temporal dimension.

### 3.5. Meta-Memory Augmentation Layer

Traffic flow data usually exhibit complex spatio-temporal characteristics, and relying solely on transient observations may not capture long-term dependencies. Therefore, to enhance the memory capacity of the neural network, a meta-memory augmentation layer is constructed between the spatio-temporal block and the output layer. This layer utilizes the attention mechanism to re-retrieve memory information. The secondary memory augmentation improves the model’s ability to recognize spatial heterogeneity, enabling it to understand relationships between traffic nodes from different perspectives and levels, and to identify hidden spatial heterogeneity. This is particularly applicable in situations where multiple traffic modes and complex interactions exist in the transport network [24,28].

**Figure 2:**
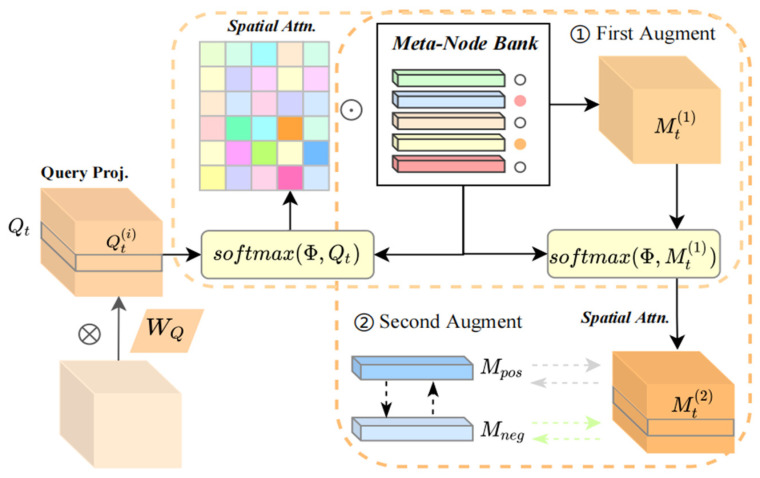
Meta-memory augmentation.

The meta-memory augmentation layer first applies a linear transformation to the outputs of the spatio-temporal blocks, generating a query projection matrix. This query is then utilized in a dual-retrieval attention mechanism, whereby it is compared against the memory units stored in the meta-memory node bank. Through this two-stage attention computation, the layer selectively retrieves and integrates relevant memory features from the meta-memory nodes. Finally, the layer fuses these two sets of augmented memory features along the channel dimension, producing the output. The processing flow is illustrated in Figure 2. 

The input spatial–temporal state is denoted as $H_{t}^{\left(i\right)}\in {\mathbb{R}}^{N\times D}$, with the meta-node bank defined previously. Given the linear weight matrix $W_{Q}\in {\mathbb{R}}^{D\times M}$ and bias $b_{Q}\in {\mathbb{R}}^{M}$, we project $H_{t}^{\left(i\right)}$ to the query matrix $Q_{t}^{\left(i\right)}$ using the equation:

(10) $$Q_{t}^{\left(i\right)}=H_{t}^{\left(i\right)}\times W_{Q}+b_{Q}$$

Subsequently, the memory similarity between the query projection and each meta-node is calculated to compute the attention scores of the retrieved information. This process identifies the historical patterns that best match the current traffic state. These attention scores are then normalized using the SoftMax function to obtain weighted memory features. The relevant equations are shown below:

(11) $$\alpha_{j}^{\left(i\right)}=\frac{\exp\left(Q_{t}^{\left(i\right)}\times \Phi^{T}\left[j\right]\right)}{\sum_{j=1}^{\phi }\exp\left(Q_{t}^{\left(i\right)}\times \Phi^{T}\left[j\right]\right)}\;$$

(12) $$M_{t,i}=\overset{\phi }{\underset{j=1}{\sum }}\alpha_{j}^{\left(i\right)}\times \Phi \left[j\right]$$

where $a_{j}^{\left(i\right)}$ denotes the similarity between the *i*-th query and the *j*-th meta-node, and *i* ∈ [1, N], j ∈ [0, M]. The resulting $M_{t}^{\left(1\right)}\in {\mathbb{R}}^{N\times D}$ represents the first memory-augmented features.

Building upon the previous step, we utilize $M_{t}^{\left(1\right)}$ as the new query projection, analogous to the formulations in Equations (11) and (12). We then compute the memory similarity weights between $M_{t}^{\left(1\right)}$ and the meta-node bank Φ, and subsequently obtain the secondary memory-augmented features $M_{t}^{\left(2\right)}$. In the final step, we perform a weighted fusion of the secondary memory-augmented features $M_{t}^{\left(1\right)}$ and $M_{t}^{\left(2\right)}$, which is then used as the output of this layer. The details are as follows:

(13) $$H_{aug}=\lambda \times M_{t}^{\left(1\right)}+\left(1-\lambda \right)\times M_{t}^{\left(2\right)}$$

Moreover, the top-2 most relevant meta-memory terms from the secondary augmented features are selected to construct positive and negative samples. During training, additional contrast loss and consistency loss are introduced to enhance the model’s ability to distinguish traffic patterns across different paths and scenarios, thereby improving the response to anomalies or unexpected events. To regulate the memory parameters, two constraints are employed [29,30] as follows:

(14) $$L_{1}=\overset{T}{\underset{t}{\sum }}\overset{N}{\underset{i}{\sum }}max\left\{{∥Q_{t}^{i},M_{pos}∥}^{2}+{∥Q_{t}^{i},M_{neg}∥}^{2}+\alpha ,0\right\}$$

(15) $$L_{2}=\overset{T}{\underset{t}{\sum }}\overset{N}{\underset{i}{\sum }}∥Q_{t}^{i},M_{pos}{∥}^{2}+\beta$$

where *T* denotes the total number of sequences. $M_{pos}$ and $M_{neg}$ denote the top 2 indices of memory items by ranking $a_{j}^{\left(i\right)}$, representing positive and negative samples, respectively. *α* denotes the margin between the positive and negative pairs, and *β* is the bias.

Here, we incorporate two additional constraints into the loss function (i.e., MAE). The overall loss for the prediction task is as follows:

(16) $$L=\overset{P,Q}{\underset{t,t^{\prime }}{\sum }}\left|{\hat{X}}_{t+t^{\prime }}-X_{t+t^{\prime }}\right|+\lambda_{1}L_{1}+\lambda_{2}L_{2}\;$$

where *λ*_1_ and *λ*_2_ are balanced *L*_1_ and *L*_2_, respectively.

## 4. Experiment

### 4.1. Experimental Setup

**Datasets.** The model presented in this paper is evaluated on four commonly used benchmark traffic datasets. All these datasets consist of aggregated samples of traffic flows at 5 min intervals. Therefore, $N_{t}=288$ (time steps in a day) and $N_{w}=7$ (days in a week) represent the lengths of the initial time embeddings. The statistical information of the datasets is shown in Table 1.

For our experiments, we preprocess the raw data samples by applying Z-score normalization to rescale the data to zero mean and unit variance. To ensure consistency with previous studies, we divided the METR-LA and PEMS-BAY datasets into training, validation, and test sets in chronological order with a ratio of 7:1:2. Similarly, for the PEMSD7(M) and PEMSD7(L) datasets, we used the first 60% of the data for training, 20% for validation, and the last 20% for testing [17]. Traffic flow data exhibit distinct time series characteristics. By partitioning the dataset based on temporal order, we can more accurately assess the model’s ability to capture long-term dependencies and short-term fluctuations in the time series data. Our objective is to predict the traffic state for the next hour based on the observations from the previous hour, i.e., *P* = 12 and *Q* = 12.

**Parameters Setup**. In the experiments conducted on all datasets, the parameters listed in Table 2 are used.

**Baselines.** To evaluate the performance of the proposed model, we compare it with several benchmark models, as shown in Table 3 and Table 4. All models are configured with the same parameter settings as those used in our experiments. We compared it against the following baseline models:

- MTGNN [5]: it utilizes adaptive graphs, mix-hop propagation layers, and dilated inception layers to capture spatial–temporal correlations.
- STGCN [14]: it combines graph convolution and one-dimensional gated convolution to capture spatial dependencies through graph convolution while using one-dimensional GRU for time series data.
- DCRNN [15]: it combines graph convolution networks with RNNs in an encoder–decoder architecture.
- GWNET [16]: Graph WaveNet introduces an adaptive adjacency matrix and combines diffuse graph convolution with TCN instead of 1D convolution.
- AGCRN [17]: it employs an adaptive adjacency graph and integrates GRU with graph convolutions with node-adaptive parameter learning.
- GMAN [31]: it is an attention-based model with spatial, temporal, and transform attention.
- VAR [32]: vector auto-regression is a classical statistical method that predicts time series data by modeling linear regression between variables, and is widely used for time series forecasting.
- FC-LSTM [33]: long short-term memory network with fully connected hidden units.

### 4.2. Performance Evaluation

Based on the results, the following observations are made: (1) Parameter Designs: The parameter designs of VAR and FC-LSTM are based on simple theoretical conditions, making it difficult to capture the complex spatial–temporal correlations in traffic data, resulting in high prediction errors. As representatives of spatial–temporal graphical models, GMAN, MTGNN, AGCRN, DSTAGNN, and MegaCRN have achieved significant performance improvements through continuous innovations in graph construction. These improvements stem from advances in integrating multiple temporal modeling components and advanced graph convolution operations. Building on existing research, our approach fully captures and exploits spatial–temporal contexts and multi-scale spatial–temporal dependencies through custom parameter learning, achieving superior prediction performance. (2) Theoretical Perspective: Our method uses rich embeddings to encode spatial–temporal context information. These embeddings capture the unique characteristics of time units and spatial states, while adaptively learning complex spatial–temporal relationships within transportation networks. This provides DSTMAN with a distinct advantage in handling these dependencies. Additionally, by combining temporal convolution and attention mechanisms, our approach effectively extracts hierarchical spatio-temporal dependencies, enabling it to handle both short-term and long-term temporal dependencies. This allows for accurate traffic flow predictions across different scales. The designed meta-node memory bank enhances the modeling of spatial heterogeneity. The secondary memory augmentation module further improves the model’s understanding of this heterogeneity. Using a retrieval attention mechanism, the model learns and utilizes information from the meta-node repository to identify hidden spatial heterogeneity across different scales and viewpoints. Across all evaluated datasets, our method improves the relative error metrics over the benchmark methods by an average of 1.2% to 2.4%.

**Figure 3:**
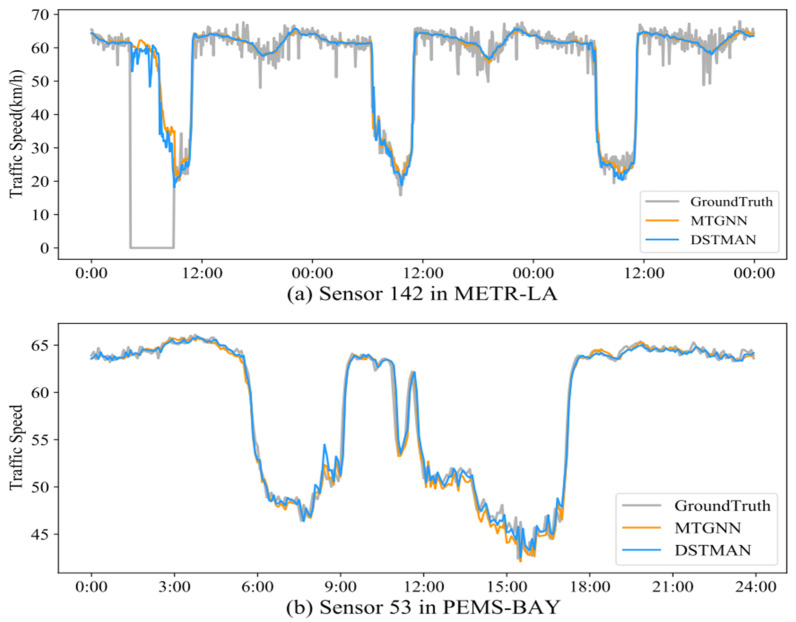
Traffic prediction visualization curves for each time within a certain day.

To visually demonstrate the difference between the predicted and real values, two nodes are selected, and comparison curves are plotted, as shown in Figure 3. By analyzing Figure 3a, it can be observed that despite the presence of noisy data, such as missing values filled with zeros, our proposed method efficiently captures the underlying historical traffic patterns, thus fitting the discontinuous missing data points well. From the analysis of the two plots, it can be seen that both our method and the sub-optimal MTGNN benchmark model effectively capture the overall evolutionary trend of traffic conditions. However, a closer inspection of the micro-curve fluctuations reveals that our method better fits the real-world conditions and exhibits a smaller error.

### 4.3. Ablation Study

To gain a deeper understanding of how individual components affect overall performance, we compare DSTMAN to four different variants of the DSTMAN framework. These variants have been evaluated on two established benchmark datasets. Specific details of the four DSTMAN variants are provided below:

- *w*/*o* DGC: It removes the diffusion graph convolution layer associated with the spatio-temporal blocks, thus eliminating part of the spatial correlation modeling. The absence of the diffusion graph convolution layer results in a reduced ability to capture spatial dependencies, where the data distribution shows significant spatial correlation, such as in the METR-LA dataset. Consequently, the model performs poorly in understanding and predicting interactions between different sensor nodes, which reduces the overall prediction performance.
- *w*/*o* DE: It replaces the data embedding layers, such as *E_d_*, *E_w,_* and *E_st_*, with fully connected networks, thus forfeiting the encoding of the spatial–temporal context. The removal of the data embedding layer resulted in the model being unable to capture temporal and spatial contextual information. This part of the design proves to be critical to model performance as it allows the model to recognize and interpret changes in different temporal and spatial states. The loss of this information significantly reduces the model’s ability to capture temporal and spatial dependencies, leading to a substantial decrease in prediction accuracy.
- *w*/*o* TC: It removes the multi-scale temporal convolutional layers from DSTMAN. The removal of the multi-scale temporal convolution layer affects the model’s ability to capture traffic patterns at different time scales. Without this layer, the model struggles to deal with temporal heterogeneity, leading to a diminished capacity to understand and predict time series data.
- *w*/*o* MA: It excludes the memory augmentation process and the computation of contrastive loss during the training phase from the architecture. Removing this module would diminish the model’s ability to capture and utilize historical information, thereby affecting its predictive performance. Additionally, the absence of this module would make the model less robust and flexible when confronted with new data.

As illustrated in Table 5, the ablation or modification of specific modules within the model architecture results in discernible performance decrements in the overall system. The substantial performance degradation incurred by the *w*/*o* DE on both datasets underscores the pivotal role of the dedicated encoding of temporal and spatial features in enabling the model to grasp the underlying spatial–temporal context. The disparate impact of *w*/*o* DGC on performance across the two datasets reflects the inherent differences in the underlying data distributions, with METR-LA exhibiting more pronounced data correlations. Moreover, the memory augmentation module (*w*/*o* MA) enhances the model’s performance beyond the capabilities afforded by the existing spatial–temporal components. Collectively, these design choices highlight the holistic and indivisible nature of DSTMAN, enabling its superior spatial–temporal forecasting performance.

### 4.4. Parameter Sensitivity Analysis

**Figure 4:**
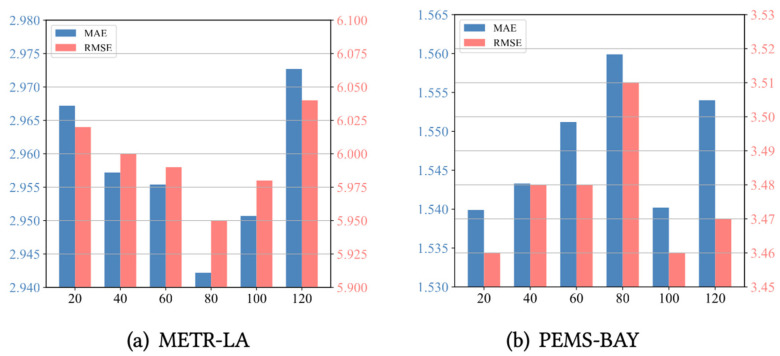
The effect of meta-memory node size.

**Figure 5:**
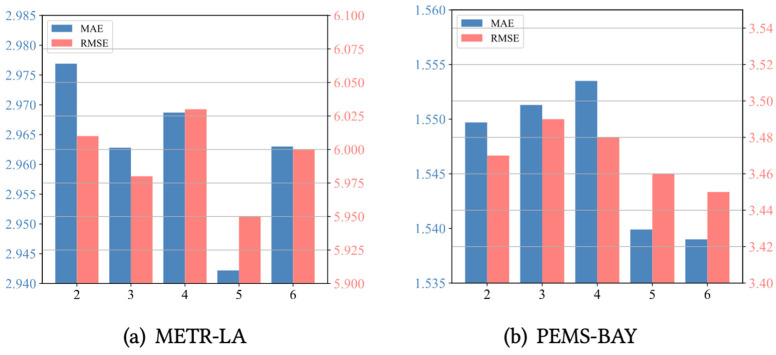
The effect of spatial–temporal block depth.

We further conducted experiments on two benchmark datasets, METR-LA and PEMS-BAY, to assess the sensitivity of the model’s key hyperparameters. This includes evaluating the effects of the meta-node bank size and spatio-temporal block depth on performance. (1) As shown in Figure 4, we analyzed the effect of the meta-node bank size on the performance of METR-LA and PEMS-BAY. We investigated the meta-node bank size within the range of 20 to 120. For the METR-LA and PEMS-BAY datasets, the optimal meta-node bank sizes are 80 and 20, respectively. Additionally, the optimal parameter for both the PEMSD7(M) and PEMSD7(L) datasets is 80. The parameter reflects the richness of the memory meta-knowledge and affects the construction of positive and negative samples in the contrastive loss calculation. An appropriate meta-node bank size can accommodate more historical information and patterns, thereby improving the model’s ability to capture complex spatio-temporal relationships. A meta-node bank that is too small may lack sufficient information, causing the model to inadequately capture complex spatio-temporal dependencies, whereas an excessively large meta-node bank may introduce redundant information, increasing computational complexity and the risk of overfitting. Therefore, it is crucial to find a balance. (2) As shown in Figure 5, model performance varies with the depth of the spatio-temporal blocks. The optimal depths for the METR-LA and PEMS-BAY datasets are 5 and 6, respectively. The depth of the spatio-temporal blocks determines the complexity of the model and its ability to capture data patterns. Shallow networks (i.e., with a small spatio-temporal block depth) may not adequately capture the complex spatio-temporal dependencies in the data, resulting in underfitting. Conversely, overly deep networks may overfit the training data, increasing computational complexity and storage burden. In general, too shallow a depth leads to underfitting, while too great a depth imposes a significant computational burden without substantial performance improvement. Consequently, in this paper, we uniformly set the network depth to 5 in the experiments to strike a reasonable balance between performance and computational burden.

### 4.5. Interpretability Analysis

**Figure 6:**
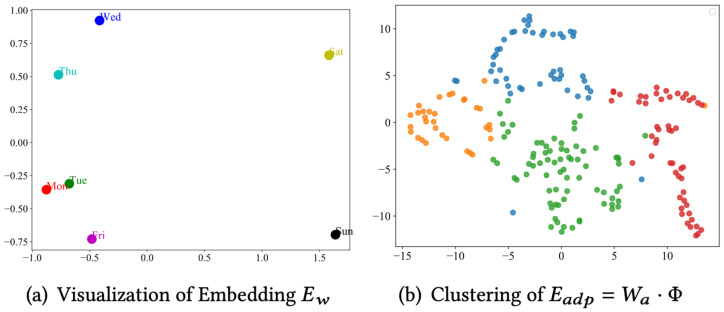
Visualization of spatial and temporal embedding.

In this section, we perform a visual analysis of the spatio-temporal embeddings learned on the METR-LA dataset to reveal the model’s performance and underlying mechanisms in traffic flow prediction. By conducting a dimensionality reduction and clustering analysis on the model’s spatio-temporal embeddings, we can gain a more intuitive understanding of how the model captures these associations. Figure 6 shows the results of a 2D visualization of a portion of the spatio-temporal embeddings, revealing the model’s ability to encode both temporal and spatial contexts. As shown in Figure 6a, weekdays and weekends are clearly separated on either side of the plane, indicating that the model effectively encodes temporal contexts, such as date types, through the $E_{w}$ component of the temporal embedding. This differentiation in the temporal context is crucial for traffic flow prediction, as traffic patterns on weekdays and weekends are usually significantly different. Figure 6b shows the clustering state of the adaptive node embeddings $E_{adp}$, which are obtained by multiplying the memory node bank with the weight matrix. The nodes can be roughly classified into four distinct clusters, with clear boundaries between different categories. Nodes within the same cluster display similar meta-knowledge. This parametric learning enables the model to identify the underlying spatial heterogeneity, which is important for traffic flow prediction because traffic patterns in different regions may vary depending on geographic location, road network structure, and other factors.

**Figure 7:**
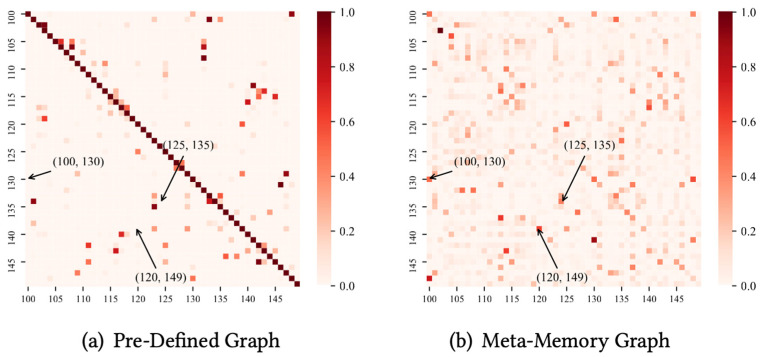
Graph structure learning.

To further analyze the spatial correlation of the model, we construct a meta-memory graph utilizing the embedding $E_{adp}$ and analyze the difference between this and the pre-defined graph in terms of latent spatial modeling through Figure 7. The color intensity of the heatmap indicates the strength of the spatial correlation. We select three pairs of nodes (100, 130), (120, 149), and (125, 135) from real traffic scenarios, which show strong associations in Figure 7b and weak associations in Figure 7a. These results indicate that meta-memory graphs effectively utilize shared and identical traffic patterns or meta-information to discover potential spatial associations, thereby compensating for the limitations of a priori knowledge. In traffic flow prediction, traditional methods often rely on pre-defined graph structures, which may not fully capture complex spatial associations. By adaptively learning potential spatial connections, the method proposed in this paper is able to more accurately reflect the dynamics in real traffic networks, thus achieving better prediction performance.

The above findings indicate that spatio-temporal embedding and adaptive node embedding play crucial roles in capturing temporal and spatial heterogeneity, identifying potential correlations, and enhancing prediction accuracy. These theoretical advantages are verified in the practical application of traffic flow prediction, providing new insights and methodologies for future research.

## 5. Conclusions

In this study, we propose a Dynamic Spatio-Temporal Memory-Augmented Network (DSTMAN) for traffic forecasting. Specifically, we first apply diverse data embeddings to encode the spatio-temporal context. Subsequently, we effectively capture the spatio-temporal correlations through stacked spatio-temporal blocks, which are constructed using multi-head temporal attention, diffusion graph convolution, and multi-scale temporal convolution modules. Finally, we introduce a novel secondary memory augmentation mechanism to enhance the model’s ability to capture spatial heterogeneity. Extensive experiments conducted on four benchmark datasets demonstrate the outstanding performance of DSTMAN. We plan to explore the application of DSTMAN to other spatio-temporal prediction tasks, such as meteorological forecasting and public health data analysis. Additionally, we aim to investigate methods to improve model robustness under extreme traffic conditions.

## Figures and Tables

**Table 1:** The overall statistics about the datasets.

Dataset	Nodes	Edges	Samples	Missing Ration
METR-LA	207	1515	34272	8.109%
PEMS-BAY	325	2369	52116	0.003%
PEMSD7(M)	228	1132	12672	0.000%
PEMSD7(L)	1026	10150	12672	0.000%

**Table 2:** Parameters of DSTMAN.

Parameters	Value
The embedding dimension *E_d_* and *E_w_*	12
The embedding dimension *E_st_*	28
The feature dimension of hidden state *d*	64
The number of attention heads	4
The order of the diffusion graph convolution	2
The number of spatial–temporal blocks	5

**Table 3:** Prediction accuracy of DSTMAN and baseline models on METR-LA and PEMS-BAY.

Dataset	Model	Horizon 3			Horizon 6			Horizon 12		
		MAE	RMSE	MAPE	MAE	RMSE	MAPE	MAE	RMSE	MAPE
METR-LA	VAR	4.42	7.89	10.20%	5.41	9.13	12.70%	6.52	10.11	15.80%
	FC-LSTM	3.44	6.30	9.60%	3.77	7.32	10.90%	4.37	8.69	13.20%
	STGCN	2.88	5.74	7.62%	3.47	7.24	9.57%	4.59	9.40	12.70%
	AGCRN	2.75	5.26	7.12%	3.13	6.29	8.59%	3.56	7.33	10.21%
	GMAN	2.80	5.55	7.41%	3.12	6.49	8.73%	3.44	7.35	10.07%
	DCRNN	2.77	5.38	7.30%	3.15	6.45	8.80%	3.60	7.60	10.50%
	GTS	2.75	5.27	7.12%	3.14	6.33	8.62%	3.59	7.44	10.25%
	STID	2.82	5.53	7.75%	3.19	6.57	9.39%	3.55	7.55	10.95%
	GWNET	2.69	5.15	6.90%	3.07	6.22	8.37%	3.53	7.37	10.01%
	MTGNN	2.69	5.18	6.86%	3.05	6.17	8.19%	3.49	7.23	9.87%
	DSTMAN	**2.65**	**5.12**	**6.80%**	**2.97**	**6.08**	**8.06%**	**3.35**	**7.07**	**9.62%**
PEMS-BAY	VAR	1.74	3.16	3.60%	2.32	4.25	5.00%	2.93	5.44	6.50%
	FC-LSTM	2.05	4.19	4.80%	2.20	4.55	5.20%	2.37	4.96	5.70%
	STGCN	1.36	2.96	2.90%	1.81	4.27	4.17%	2.49	5.69	5.79%
	AGCRN	1.37	2.87	2.94%	1.69	3.85	3.87%	1.96	4.54	4.64%
	GMAN	1.35	2.93	2.84%	1.66	3.79	3.68%	1.91	4.43	4.39%
	DCRNN	1.38	2.95	2.90%	1.74	3.97	3.90%	2.07	4.74	4.90%
	GTS	1.37	2.92	2.85%	1.72	3.86	3.88%	2.06	4.60	4.88%
	STID	1.31	2.79	2.78%	1.64	3.73	3.73%	1.91	4.42	4.55%
	GWNET	1.30	**2.74**	2.73%	1.63	3.70	3.67%	1.95	4.52	4.63%
	MTGNN	1.32	2.79	2.77%	1.65	3.74	3.69%	1.94	4.49	4.53%
	DSTMAN	**1.30**	2.76	**2.72%**	**1.60**	**3.68**	**3.57%**	**1.86**	**4.32**	**4.30%**

**Table 4:** Prediction accuracy of DSTMAN and baseline models on PEMSD7(M) and PEMSD7(M).

Dataset	Metric	STGCN	DCRNN	AGCRN	GWNET	MTGNN	DSTAGNN	ST-WA	MegaCRN	DSTMAN
PEMSD7(M)	MAE	2.66	3.07	2.76	2.63	2.65	2.78	2.67	2.57	**2.56**
	RMSE	5.38	6.34	5.57	5.32	5.45	5.54	5.36	5.28	**5.10**
	MAPE	6.65%	7.56%	6.97%	6.66%	6.47%	6.93%	6.66%	**6.37%**	6.38%
PEMSD7(L)	MAE	2.94	3.30	2.95	2.95	2.82	2.98	2.94	2.86	**2.78**
	RMSE	5.91	5.98	5.95	5.85	5.80	6.43	5.92	5.80	**5.57**
	MAPE	7.36%	8.16%	7.47%	7.47%	7.15%	7.50%	7.51%	7.23%	**7.04%**

**Table 5:** Ablation experiments of DSTMAN.

Dataset	Model and Variants	MAE	RMSE	MAPE
METR-LA	*w*/*o* DGC	3.12	6.46	8.97%
	*w*/*o* DE	3.08	6.15	8.18%
	*w*/*o* TC	3.01	6.09	8.24%
	*w*/*o* MA	2.98	6.06	8.17%
	DSTMAN	**2.94**	**5.95**	**7.98%**
PEMS-BAY	*w*/*o* DGC	1.57	3.54	3.55%
	*w*/*o* DE	1.59	3.53	3.55%
	*w*/*o* TC	1.57	3.51	3.50%
	*w*/*o* MA	1.55	3.50	3.45%
	DSTMAN	**1.54**	**3.46**	**3.41%**

## Data Availability

The datasets used in this paper are all publicly available. METR-LA, PEMSBAY, PEMSD7(M), and PEMSD7(L) datasets are derived from the California Department of Transportation’s (Caltrans) Performance Measurement System (PeMS) for traffic monitoring. Further inquiries can be directed to the corresponding author.
